# Autogenous Tooth Bone Grafts with Enamel Matrix Derivates in Non-Contained Intrabony Periodontal Defects—A Case Series Study

**DOI:** 10.3390/biomedicines14010056

**Published:** 2025-12-26

**Authors:** Eleonora Solyom, Kristóf Forgó, Kristof Somodi, Daniel Palkovics, Szilard Vancsa, Peter Windisch, Balint Molnar, Reka Fazekas

**Affiliations:** 1 Department of Periodontology, Semmelweis University, 1088 Budapest, Hungary; 2 Centre for Translational Medicine, Semmelweis University, 1085 Budapest, Hungary; 3 Institute for Translational Medicine, Medical School, University of Pécs, 7622 Pécs, Hungary; 4 Institute of Pancreatic Diseases, Semmelweis University, 1083 Budapest, Hungary; 5 Department of Restorative Dentistry and Endodontics, Semmelweis University, 1085 Budapest, Hungary

**Keywords:** autogenous tooth graft, enamel matrix derivative, periodontal regeneration, intrabony defect, bone substitute

## Abstract

**Background:** The predictability of regenerative outcomes in non-contained intrabony periodontal defects remains limited. Autogenous tooth bone grafts (ATB) may represent a biologically active and osteoconductive scaffold with minimal residual graft material. This study evaluated the clinical and radiographic outcomes of ATB combined with enamel matrix derivative (EMD) in intrabony defects. **Methods:** Nine systemically healthy patients (15 defects) were treated with ATB + EMD in a retrospective proof-of-concept design. Clinical parameters—probing pocket depth (PPD), clinical attachment level (CAL), and gingival recession (GR)—were recorded at baseline and 6 months. Radiographic changes in defect depth and width were also assessed. Statistical significance was set at *p* < 0.05. **Results:** Mean PPD decreased from 7.73 ± 0.96 mm to 3.87 ± 0.74 mm (*p* < 0.001), and CAL improved from 9.20 ± 1.47 mm to 5.53 ± 1.36 mm (*p* < 0.001). GR changes were not significant. Radiographically, mean defect depth and width were reduced from 3.81 ± 1.59 mm and 2.56 ± 0.75 mm to 0.72 ± 1.08 mm and 0.44 ± 0.70 mm, respectively (*p* < 0.001). **Conclusions:** The combination of ATB and EMD yielded substantial clinical and radiographic improvements in intrabony periodontal defects. These findings suggest that autogenous tooth bone grafts may serve as a reliable biologically active scaffold for regenerative periodontal surgery. This is the first study evaluating the combination of EMD and ATB. Within the study limitations, ATB + EMD demonstrated promising regenerative potential, warranting future controlled clinical trials.

## 1. Introduction

One of the main challenges in periodontal reconstructive surgery is the management of non-contained intrabony defects, in which one or more bony walls are missing. These defects provide insufficient natural containment, compromising blood clot stability and space maintenance, and consequently reducing their regenerative potential compared with well-contained defects [1]. Enamel matrix derivative (EMD) has been histologically proven to support periodontal regeneration by promoting new cementum and alveolar bone formation [2,3,4,5]. While EMD is effective in contained defects, its application in non-contained intrabony defects is often insufficient due to inadequate blood clot stabilization. In such cases, even with the use of microsurgical techniques, combining EMD with a particulate scaffold is typically required to achieve predictable outcomes [6].

According to data from the American Academy of Periodontology (AAP), the use of combination therapies involving biologically active materials can enhance regenerative outcomes [7]. A recent meta-analysis demonstrated that combining EMD with a graft material resulted in superior outcomes in terms of gingival recession (GR) and defect fill compared to EMD alone [8]. Another meta-analysis reported significantly greater improvements in probing pocket depth (PPD) reduction and clinical attachment level (CAL) gain when EMD was used in combination with a graft material versus EMD alone [9].

Among particulate grafts, autogenous bone is considered the gold standard due to its osteogenic potential [10]. However, its clinical use is limited by rapid resorption, limited availability, and the morbidity associated with harvesting procedures. Consequently, the focus has shifted towards alternative graft materials, such as xenografts, allografts, and synthetic substitutes. Despite their widespread use, these materials often demonstrate limited remodeling capacity and can leave a significant amount of residual graft particles. Reports indicate residual particle rates of up to 42%, with new bone formation as low as 30%, which may contribute to late complications and hinder long-term regeneration [11,12,13,14].

Autogenous tooth bone grafts (ATBs), processed from extracted teeth, have recently emerged as a promising patient-derived alternative to conventional grafting materials. Their inorganic hydroxyapatite content and organic matrix—rich in type I collagen and bioactive molecules such as BMPs, TGF-β, and IGF-1—closely resemble the composition of alveolar bone, providing a biologically compatible scaffold for regeneration. Since their clinical introduction in 2008, ATBs have been successfully applied in socket preservation and guided bone regeneration procedures [15].

Supporting this concept, Pohl et al. demonstrated superior clinical outcomes when autologous dentin grafts were used for socket preservation in patients treated with the socket-shield technique. In their retrospective case series, sites augmented with dentin grafts showed reduced soft tissue ingrowth, improved maintenance of ridge contour, and predictable conditions for subsequent implant placement compared with sites treated without autogenous tissue grafts [16]. These findings further suggest that autogenous dentin-based materials may offer biological and clinical advantages over conventional xenografts. Notably, ATBs demonstrate substantially lower residual graft proportions (9–18%) compared with xenogenic and allogenic substitutes (40–54%), indicating a more favorable remodeling profile [15,17,18].

ATB used as a sole grafting material may offer limited periodontal regenerative potential, as it lacks biologic mediators required to induce cementoblast differentiation or fibroblast activation for Sharpey fiber formation. In contrast, combining ATB with EMD may provide complementary benefits: ATB offers a stable, space-maintaining scaffold, whereas EMD supplies the bioactive proteins necessary to support true periodontal regeneration. Enamel matrix derivative is a well-established and clinically validated biologic with strong evidence supporting its efficacy in promoting periodontal regeneration. ATB, however, has only been reported in isolated case reports included only three patients within periodontal defects, only in furcation-involved teeth, and its broader applicability in intrabony defect reconstruction has not been evaluated [19,20].

Although both materials show individual potential [21,22], evidence regarding their combined use in intrabony periodontal defects is scarce, and have not been explored their efficacy in this context. Therefore, this retrospective proof-of-concept study aimed to evaluate the clinical and radiographic outcomes of ATB combined with EMD for the regenerative treatment of non-contained intrabony periodontal defects. We hypothesized that this biologically enhanced combination would improve clinical attachment gain and radiographic bone fill compared with baseline values, supporting its potential as a predictable alternative for periodontal regeneration.

## 2. Materials and Methods

The study was approved by the local ethical committee at Semmelweis University, Budapest, Hungary (SE RKEB: 116/2023) and was conducted following the declaration of Helsinki, as revised in 2013 [23]. Surgical interventions were performed with the understanding and written informed consent of each participant. This case series has been reported in line with the PROCESS Guideline [24].

### 2.1. Study Design

This retrospective, proof-of-concept, single-centre case series study recruited participants from October 2023 to January 2024 at the Department of Periodontology, Semmelweis University, Hungary.

### 2.2. Participants

Nine healthy periodontal patients (three males, six females, mean age 35) with ≥4 mm deep intrabony defects and hopeless teeth for extraction were selected. Patients presenting with multiple defects were also included; however, only one defect per quadrant—specifically the site exhibiting the greatest probing pocket depth—was selected for analysis. Given the retrospective design of the study, all non-contained intrabony periodontal defects (one- and two-wall defects) presenting with an intrabony component of at least 4 mm and treated with ATB in combination with EMD were included. The hopeless tooth, which was extracted to obtain the graft material, had to be located in a different quadrant from the one selected for surgical treatment. Exclusion criteria were the following: (1) significant medical conditions: non-controlled diabetes mellitus [25], irradiation in the maxillofacial area, uncontrolled diabetes or hypertension, systemic steroid or bisphosphonate treatment, pregnancy, or lactation, infectious diseases, (2) age < 18 years, (3) smokers (>5 cigarettes/day), (4) high residual inflammation (FMBS > 25%) [26], (5) poor oral hygiene (FMPS > 25%) [26], and (6) defect-related factors: furcation involvement, endo-perio defects, horizontal defects, or multi-tooth defects.

### 2.3. Intervention Details

All patients received initial cause-related periodontal therapy, followed by comprehensive clinical and radiographic assessment prior to surgery. The initial treatment phase consisted of supra- and subgingival scaling and root surface debridement, followed by re-evaluation eight weeks later. The coexistence of intrabony defects and teeth with a hopeless prognosis for extraction has allowed patients to undergo regenerative periodontal therapy with ATB, supplemented by the application of Emdogain^®^ (EMD, Straumann, Basel, Switzerland).

### 2.4. Preparation of the ATB

**Figure 1:**
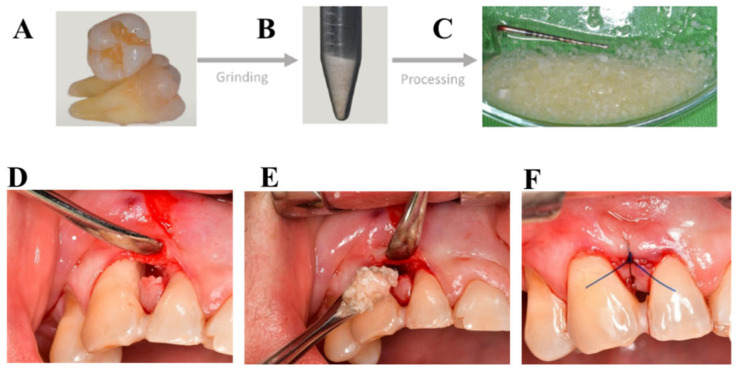
Autogenous tooth bone graft processing method and surgical steps. (**A**) Cleaned tooth after extraction. (**B**) ATB after the grinding process. (**C**) ATB after processing procedures. (**D**) Flap elevation. (**E**) Filling ATB into the vertical defect. (**F**) Vertical mattress sutures for closure.

After local anesthesia (Ultracain DS forte, Sanofi-Aventis, Paris, France), the tooth with a hopeless prognosis was extracted on the day of surgery and prepared according to the manufacturer’s recommendations. The tooth used for graft preparation was always extracted from a different quadrant than the surgical site to avoid any interference with postoperative healing. After completing meticulous debridement, we manually pre-fractured the teeth into coarse fragments using a hammer-and-pestle, which facilitated subsequent standardized grinding. After the grinding the particles sized 425–1500 µm were used. Demineralization was carried out using the BonMaker^®^ (Meta BioMed Co., Ltd., Cheongju, Republic of Korea) system according to the manufacturer-recommended automated protocol. For particulate graft preparation, the device applied sequential chemical treatments, including ~0.45 M hydrochloric acid, high-volume hydrogen peroxide, ethanol, chloroform, and final saline rinses [27] (Figure 1).

### 2.5. Surgical Regenerative Procedure

At the site of the periodontal procedure, a full thickness of mucoperiosteal flap was raised under local anesthesia (Ultracain DS forte, Sanofi-Aventis). According to the mesiodistal width of the interproximal space, a modified- or simplified papilla preservation technique (MPPT and SPPT) [28,29] was utilized to access the intrabony defect. After removing granulation tissue, scaling, and root planning were conducted using Gracey curettes and ultrasonic devices. The debrided root surfaces were conditioned with 24% ethylenediamine-tetraacetic acid (EDTA) gel for 2 min, followed by a one-minute rinse with sterile saline solution. After drying the root surface, EMD was applied on the root surface and also at the ATB. After 20 s, ATB was placed into the intrabony defect. Finally, the mucoperiosteal flap was adapted and primary, tension-free closure was achieved with vertical or horizontal mattress sutures using a 6-0 monofilament suturing material (Chiraflon, Vitrex MedicalA/S, Herlev, Denmark) (Figure 1).

### 2.6. Postsurgical Care

Surgeries were performed by two experienced periodontists (E.S. and K.F.). Postoperatively, patients were instructed to rinse with 0.12% chlorhexidine-digluconate two times a day for 2 weeks. Participants were advised to avoid mechanical teeth cleaning at the surgical sites and interproximal area. Sutures were removed 14 days after the surgery.

### 2.7. Outcome Measures

#### 2.7.1. Clinical Assessment

Baseline clinical parameters were recorded after non-surgical therapy; before surgery (T0), and 6 months postoperatively (T1) using a UNC-15 probe at each affected tooth and two neighboring teeth. Two independent examiners (K.S. and D.P.), uninvolved in surgery, measured six sites per tooth: mesio-buccal (mb), mid-buccal (b), disto-buccal (db), mesio-lingual (ml), mid-lingual (l) and disto-lingual (dl).∙The following parameters were recorded at T0 and T1: (i) clinical attachment level (CAL): distance in millimeters from the cemento-enamel junction (CEJ) to the bottom of the pocket; (ii) probing pocket depth (PPD): distance in millimeters from the gingival margin to the bottom of the pocket; (iii) gingival recession (GR): distance in millimeters from the gingival margin to the cemento-enamel junction (CEJ); (iv) presence or absence of dental plaque (PI); (v) presence or absence of bleeding on probing (BoP). In addition, FMPS (percentage of tooth sites revealing the presence of dental biofilms) and FMBS (percentage of tooth sites revealing the presence of bleeding on probing) were also recorded.

#### 2.7.2. Radiographical Assessment

**Figure 2:**
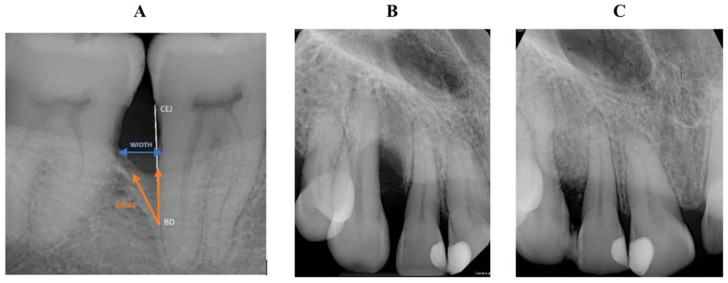
(**A**) Radiographical measurement parameters, showing cemento-enamel junction (CEJ), the width (WIDTH); the base (BD), and the angulation (ANGLE) of the periodontal defect; (**B**) Preoperative radiography showing the one-wall bony defect (**C**) postoperative radiography taken after six months. Radiographically, no periodontal defect can be detected.

Intraoral radiographs (IRs) were taken before surgery (T0) and at 6 months (T1) using a parallel cone technique and analyzed with radiographic software (IMPAX, Agfa, v. 3.1.1. Mortsel, Belgium). On the pre- and postoperative IRs, a 10 mm line markup was placed outside the surgical area as a reference line. Images were further processed using the analysis platform FIJI-ImageJ (https://imagej.net). On the radiographs, the depth (INTRA) and width (WIDTH) of the intrabony component and the radiographic defect angle (ANGLE) were measured by two blinded and calibrated investigators with radiological expertise (K.S. and D.P.) (Figure 2). In case of discrepancy, a third examiner assessed the X-rays (B.M.). Results at T0 and T1 were compared.

### 2.8. Statistical Analysis

All statistical calculations were performed with R (R Core Team 2020, v4.0.3). Descriptive statistics were used to describe all variables as mean ± standard deviation. No missing data were available. Statistical significances were assessed by inferential statistics with an α = 0.05 significance level. The normality of the examined variables was checked analytically with the Shapiro–Wilk test and graphically with a quantile-quantile plot for each variable.

Clinical variables (PPD, CAL, GR) were found to be normally distributed. Hence, a paired *t*-test assessed statistical differences between T0 and T1. Regarding radiographic parameters (INTRA, WIDTH, ANGLE), distributional assumptions were not satisfied. Therefore, Wilcoxon matched-pairs sign-rank tests were utilized to assess each variable’s statistical significance between T0 and T1 values. Results were graphically represented using box plots.

## 3. Results

### 3.1. Patient Characteristics

After identification and screening, 9 patients fulfilling the inclusion criteria were treated. Finally, data from 9 patients with 15 defects were collected and used for analysis in the present study. All patients were generally healthy. The mean age was 35 ± 5.4 years. Six patients were female, and three patients were male. Three patients were considered light smokers (<5 cigarettes/day). All patients underwent initial periodontal therapy.

Six defects were located in the mandible and nine in the maxilla. Eight teeth were single-rooted, five premolars, three front teeth, and seven molars were included. Eight intrabony defects presented one-wall morphology, and another seven defects showed two-wall morphology (Table 1).

### 3.2. Clinical Outcomes

**Figure 3:**
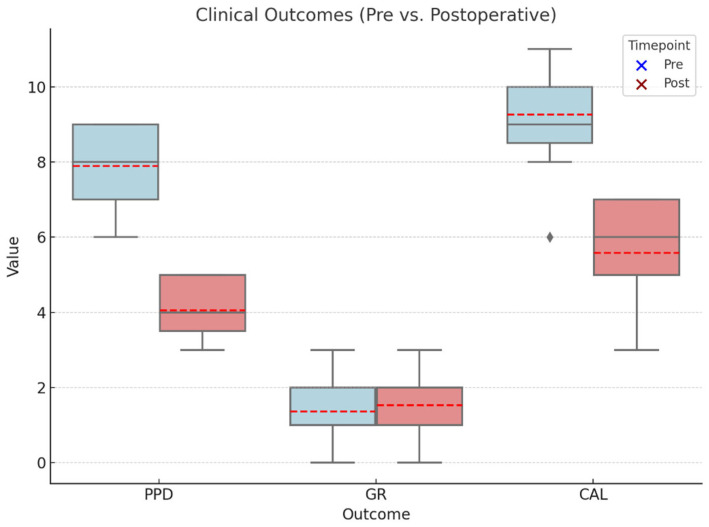
Boxplots representing the clinical outcomes: pre- and postoperative (6 months) probing pocket depth (PPD), gingival recession (GR), and clinical attachment loss (CAL). Red lines representing the mean values.

At all of the treated tooth site the healing was eventless, no complications were detected. The study’s primary outcome was the PPD change between T0 and T1. PPD values at the deepest point of the defect averaged at 7.73 mm ± 0.96 mm and 3.87 mm ± 0.74 mm at T0 and T1, respectively. The change was statistically significant (*p* < 0.001). At the same measurement site, GR values were found to be 1.47 mm ± 0.99 mm at T0 and 1.66 mm ± 0.82 mm at T1, which change was found to be statistically non-significant (*p* = 0.19). CAL values averaged 9.20 mm ± 1.47 mm and 5.53 mm ± 1.36 mm at T0 and T1, respectively. A statistically significant change was detected between T0 and T1 CAL values (*p* < 0.001). Box plots in Figure 3 show the distribution of baseline and 6-month follow-up clinical data. Clinical data are summarized in Figure 3.

Looking at the individual defects, we could see an improvement in all cases regarding PPD and CAL. Regarding GR, subtle clinically insignificant variations were observed (Table 2).

The frequency distribution of clinical outcomes is shown in Table 3. The majority of defects demonstrated marked improvement, with most sites achieving a PPD reduction and CAL gain of 4–5 mm at six months.

The frequency distribution of clinical attachment level (CAL) gain at six months is presented in Table 4. Most treated sites showed substantial clinical improvement, 66.7% of defects achieved a gain of 4–5 mm, while only one site (6.7%) showed a gain of ≤1 mm.

### 3.3. Radiographic Outcomes

**Figure 4:**
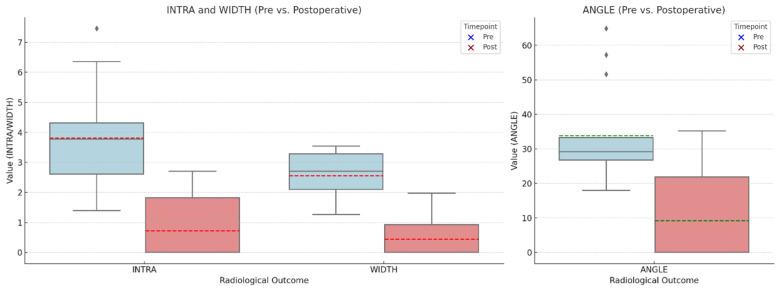
Clinical outcomes of preoperative and postoperative (6 months) depth; width and angle of intrabony components. Red and green lines represents the mean values.

Dimensions of intrabony defects (INTRA, WIDTH, ANGLE) were assessed with linear measurements on IR with a calibration line (Figure 4). The inter-observer agreement was 94%.

Mean INTRA was measured to be 3.81 mm ± 1.59 mm at T0 and 0.72 mm ± 1.08 mm at T1. This difference was statistically significant using the Wilcoxon matched-pairs sign-rank test (*p* < 0.001). WIDTH was measured to be 2.56 mm ± 0.75 mm and 0.44 mm ± 0.70 mm at T0 and T1, respectively, which change was statistically significant (*p* < 0.001). ANGLE changed from 33.83° ± 13.38° at T0 to 9.15° ± 13.83° at T1, which difference was found to be statistically significant (*p* = 0.0012). Box plots in Figure 4 show the distribution of baseline and 6-month follow-up clinical data. Radiographic data are summarized in Table 5.

## 4. Discussion

Periodontal reconstructive therapy of non-contained intrabony defects remains a significant challenge in periodontal therapy. This retrospective study evaluated the efficacy of ATB with EMD for periodontal regeneration in one- and two wall intrabony defects. ATB offers several advantages due to its natural composition, which resembles bone and provides a scaffold for new bone formation. Additionally, ATB contains growth factors and bioactive molecules that stimulate osteogenic activity, while EMD has chemotactic effects on various cell types involved in wound healing and tissue regeneration [30].

Enamel matrix derivative (EMD) is a well-researched and extensively documented regenerative biomaterial with extensive clinical evidence supporting its efficacy in promoting periodontal healing and improving clinical outcomes [3,31,32,33].

In the present case series, the combination of ATB and EMD resulted in a mean PPD reduction of 3.86 mm and a mean CAL gain of 3.67 mm after six months. These outcomes fall within the range reported for EMD used in combination with other particulate graft materials. For example, Yilmaz et al. reported a CAL gain of 4.2 mm and a PPD reduction of 4.0 mm using EMD with autogenous bone grafts [34], while Sculean et al. observed a PPD reduction of 5.5 mm when EMD was combined with a xenograft [35]. The magnitude of improvement in the present cohort therefore falls within the range of clinical outcomes achieved in previous studies applying particulate grafts alongside EMD. However, due to the descriptive nature of our study and the absence of a control group, these comparisons should be interpreted with caution.

Beyond mean values, the frequency distribution of clinical outcomes demonstrated a consistent regenerative response across most treated defects. As shown in Table 3 and Table 4, the majority of sites achieved a PPD reduction and CAL gain of 4–5 mm at six months, despite the inclusion of exclusively non-contained one- and two-wall intrabony defects. This homogeneous distribution suggests a predictable clinical response in a defect morphology traditionally associated with reduced regenerative potential. These findings may be interpreted in the context of the systematic review by Aimetti et al., which evaluated pocket resolution following regenerative treatment of intrabony defects using papilla preservation techniques. That analysis identified final probing depth thresholds of ≤3 mm and ≤4 mm as clinically meaningful endpoints and reported weighted mean pocket resolution rates of 61.4% and 92.1%, respectively, at 12 months [36]. Although the present study did not assess pocket resolution as a dichotomous outcome, the magnitude and distribution of PPD reduction observed—together with a mean postoperative PPD of 3.87 mm—suggest that a substantial proportion of treated sites likely approached the PD ≤4 mm threshold considered compatible with periodontal stability and long-term maintainability. Importantly, Aimetti et al. emphasized the importance of biologically driven regeneration combined with adequate wound stability and space maintenance.

Previous studies support the concept that combining EMD with a particulate graft enhances the regenerative environment compared with EMD alone in certain defect morphologies. Cochran et al. demonstrated greater cementum and bone formation when EMD was combined with autogenous bone grafts compared with EMD alone [37]. Although recent meta-analyses have not shown statistically significant differences between EMD monotherapy and combination therapy, mean CAL gain consistently favored combined approaches [33]. These observations support the rationale for investigating ATB as an adjunctive scaffold in periodontal regeneration.

Existing clinical evidence suggests that the combination of EMD with a particulate graft may enhance the stability of the regenerative environment compared with the use of EMD alone in certain defect morphologies. On this basis, the present study aimed to investigate the adjunctive use of an autogenous tooth-derived graft (ATB) in conjunction with EMD in the treatment of intrabony periodontal defects. To date, no case reports or controlled clinical studies have evaluated the clinical performance of ATB combined with EMD in periodontal reconstructive procedures.

Clinical parameters were assessed at baseline and six months post-surgery. Each case demonstrated a significant decrease in mean pocket depth and gingival recession. In case of one or two-wall bony defects despite the minimally invasive flap designs, the blood-clot stability is not sustainable, particulate graft materials are needed. However conventionally used particulate graft materials only have osteoconductive properties. With the combination of ATB and EMD a biologically enhanced healing process and predictable defect fill may be expected.

In 1982, Movin & Borring-Møller explored the effectiveness of allogenic demineralized dentin in treating 14 infrabony periodontal defects. After a twelve-month follow-up, no clinical signs of dentin implant rejection were observed, and there were no statistically significant differences in PD between the test and control groups. However, delayed soft tissue healing was noted, likely due to the lack of a standardized protocol for demineralized dentin graft preparation at the time [38].

In 2021, Dhuvad et al. conducted a study using dentin grafts in over 200 procedures, including alveolar bone grafting, sinus grafting, and socket preservation. Dehiscence of the graft was only observed in 5 cases, and X-rays revealed a dense dentin-bone composite. They concluded that autogenous mineralized dentin particulate grafts could be effectively used for dental regeneration [39].

A split-mouth pilot study by Luis Sánchez-Labrador et al. reported the use of autologous dentin graft to prevent periodontal defects distal to the second molar after third molar extraction. After six months, PD decreased from 6.48 ± 1.15 mm to 3.98 ± 0.79 mm, with a radiographically observed mean bone fill [40].

Elsherbini et al. (2023) [41] examined the efficacy of tooth graft material in regenerating periodontal vertical alveolar defects on albino rats. After six months, they observed a notable reduction in probing pocket depth (PPD) from 5.07 ± 0.6 mm to 2.64 ± 0.4 mm, and clinical attachment level (CAL) decreased from 5.33 ± 0.32 mm to 3.16 ± 0.5 mm. These findings are similar to our study, demonstrating the efficacy of tooth graft material in promoting periodontal regeneration [41].

Numerous In Vitro, animal, and clinical studies have consistently confirmed the biocompatibility, biodegradability, and potent osteoinductive and osteoconductive properties of the dentin matrix. Histological examinations have shown the gradual resorption of autogenous DDM, which is typically replaced by new bone around six months post-grafting [14,17,18,30,42,43]. Finkelman et al. concluded that dentin and bone have similar chemical compositions as mineralized tissues. Both Demineralized Dentin Matrix (DDM) and Demineralized Bone Matrix (DBM) primarily consist of type 1 collagen (approximately 95%) and non-collagenous proteins, including a small proportion of growth factors. These findings highlight the significant potential of DDM as a practical biomaterial for enhancing bone regeneration in clinical settings [44].

Various case reports have demonstrated the use of ATB for periodontal regeneration, showing promising results in terms of pocket depth reduction, clinical attachment level gain, and bone fill (both linear and percentage) [20,41,45,46].

Despite the encouraging outcomes, this study has several limitations. The small sample size and retrospective design limit generalizability. Radiographic measurements, while standardized and reproducible, cannot provide histological confirmation of true periodontal regeneration. This study is descriptive in nature and, as such, cannot determine whether ATB offers any additional benefit beyond EMD used alone or minimally invasive surgical therapy. Moreover, short-term follow-up (6 months) restricts conclusions on long-term stability. Future studies should include larger, randomized controlled trials with histological and 3D imaging analyses to confirm bone maturation, as well as standardized evaluation of inter- and intra-observer reliability.

## 5. Conclusions

The combination of ATB and EMD offers a biologically favorable, autogenous alternative to conventional particulate grafts in regenerative periodontal surgery. This approach may reduce the risk of foreign body reactions, minimize residual graft remnants, and accelerate tissue remodeling. Additionally, as the donor tooth is extracted for therapeutic reasons, ATB eliminates the need for secondary bone harvesting procedures, thus lowering patient morbidity and cost.

Within the limitations of this retrospective proof-of-concept study, the combination of autogenous tooth bone grafts (ATB) and enamel matrix derivative (EMD) resulted in significant clinical attachment gain, probing pocket depth reduction, and radiographic bone fill in non-contained intrabony periodontal defects. These outcomes highlight ATB as a biocompatible, patient-derived scaffold capable of supporting periodontal regeneration when combined with biologically active agents such as EMD.

## Figures and Tables

**Table 1:** Baseline patient and defect characteristics.

Variable	Value/Description
Number of patients (*n*)	9
Number of treated defects (*n*)	15
Age (years, mean ± SD)	35 ± 5.4
Sex (M/F)	3/6
Smokers (<5 cigarettes/day)	3 (33.3%)
Systemic health	All patients generally healthy
Defect location	Maxilla: 9; Mandible: 6
Tooth type	Single-rooted: 8; Multi-rooted: 7
Defect morphology	One-wall: 8; Two-wall: 7
Full-mouth plaque score (FMPS, %)	<25
Full-mouth bleeding score (FMBS, %)	<25
Follow-up period (months)	6

**Table 2:** Table represents the mean, standard deviation, standard error, confidence interval, minimum, median, and maximum for T0 and T1 measurement and the paired *t*-test.

	PPD		GR		CAL	
	Baseline (T0)	6-Month Follow-Up (T1)	Baseline (T0)	6-Month Follow-Up (T1)	Baseline (T0)	6-Month Follow-Up (T1)
Mean	7.73	3.87	1.47	1.67	9.20	5.53
SD	0.96	0.74	0.99	0.82	1.47	1.36
SE	0.25	0.19	0.26	0.21	0.38	0.35
Conf. int.	7.20–8.27	3.46–4.28	0.92–2.02	1.21–2.12	8.38–10.02	4.78–6.28
Minimum	6	3	0	0	6	3
Maximum	9	5	3	3	11	7
Median	8	9	2	2	9	6
*p*-value	<0.001		0.19		<0.001	

**Table 3:** Frequency distribution of clinical improvements in PPD and CAL at 6-month follow-up in millimeter.

Ref. no.	PPD			CAL		
	Pre	Post	Change	Pre	Post	Change
1	8	4	4	11	7	4
2	6	3	3	6	3	3
3	9	5	4	11	7	4
4	7	5	2	8	7	1
5	7	4	3	9	6	3
6	8	4	4	11	7	4
7	9	3	6	9	4	5
8	8	3	5	8	4	4
9	9	4	5	10	6	4
10	7	3	4	8	4	4
11	7	4	3	8	5	3
12	8	4	4	10	5	5
13	7	3	4	9	5	4
14	7	4	3	9	6	3
15	9	5	4	11	7	4
Mean ± st.dev.	7.73 ± 0.96	3.87 ± 0.74	3.87 ± 0.99	9.20 ± 1.47	5.53 ± 1.35	3.67 ± 0.98
Median	8	4	4	9	6	4

**Table 4:** Clinical attachment level gain frequency distribution in percentage.

CAL Gain Frequency Distribution		
	Count	Percentage
0–1 mm	1	6.67
2–3 mm	4	26.67
4–5 mm	10	66.67
6 mm <	0	0

**Table 5:** Table represents the mean, standard deviation, minimum, median, and maximum for T0 and T1 measurement and the paired *t*-test.

	INTRA ^2^ (mm)		WIDTH ^3^ (mm)		ANGLE ^4^ (^o^)	
	Baseline (T0)	6-Month Follow-Up (T1)	Baseline (T0)	6-Month Follow-Up (T1)	Baseline (T0)	6-Month Follow-Up (T1)
Mean (SD)	3.81 (1.59)	0.72 (1.08)	2.57 (0.75)	0.45 (0.70)	33.83 (13.38)	9.15 (13.83)
Median	3.78	0.00	2.71	0.00	29.19	0.00
Minimum	1.40	0.00	1.27	0.00	17.97	0.00
Maximum	7.46	2.71	3.54	1.98	64.86	35.19
*p*-value ^1^	<0.001		<0.001		<0.001	

^1^ Wilcoxon matched-pairs sign-rank test (α = 0.05). ^2^ depth of the intrabony component. ^3^ width of the intrabony component. ^4^ radiographic defect angle.

## Data Availability

The original contributions presented in this study are included in the article. Further inquiries can be directed to the corresponding author.

## Abbreviations

AAP	American Academy of Periodontology
ANGLE	Radiographic defect angle
ATB	Autogenous Tooth Bone (graft)
BD	Base of the defect
BMC	Bone Mineral Content (contextual general meaning)
CAL	Clinical Attachment Level
CEJ	Cemento-Enamel Junction
DBM	Demineralized Bone Matrix
DDM	Demineralized Dentin Matrix
DS	Dose Strength (context: Ultracain DS Forte)
EDTA	Ethylenediaminetetraacetic Acid
EMD	Enamel Matrix Derivative
FIJI	FIJI ImageJ Analysis Software
FMBS	Full-Mouth Bleeding Score
FMPS	Full-Mouth Plaque Score
GR	Gingival Recession
IGF	Insulin-like Growth Factor
IMPAX	Radiographic Analysis Software
INTRA	Intrabony defect depth
IR	Intraoral Radiograph
MPPT	Modified Papilla Preservation Technique
PD	Pocket Depth
PI	Plaque Index
PPD	Probing Pocket Depth
RKEB	Regional Institutional Ethics Committee (Semmelweis University)
SE	Semmelweis University
SPPT	Simplified Papilla Preservation Technique
TGF	Transforming Growth Factor
UNC	University of North Carolina (periodontal probe)
WIDTH	Width of the intrabony defect
